# The Association Between Body Mass Index and Parental History of Hypertension Among Young Indian Adults

**DOI:** 10.7759/cureus.40670

**Published:** 2023-06-19

**Authors:** Barkha Jain, Raghvendra Gumashta, Jyotsna Gumashta, Rohan Garg, Vinu Vij

**Affiliations:** 1 Obstetrics and Gynaecology, Atal Bihari Vajpayee Government Medical College, Vidisha, IND; 2 Community Medicine, People's College of Medical Sciences and Research Centre, Bhopal, IND; 3 Physiology, All India Institute of Medical Sciences (AIIMS), Nagpur, IND; 4 General Medicine, Netaji Subhash Chandra Bose Medical College, Jabalpur, IND

**Keywords:** asian indian, body mass index, parental history of hypertension, primary prevention, youth health

## Abstract

Background: Hypertension is a disease of multifactorial etiology. Individuals with parental history of hypertension (PHH) have also been observed to be at an increased risk of developing hypertension in their adult life. However, there is a dearth of studies among youth. Obesity is one of the risk factors, and studies conducted among adults of all age groups have observed that there is a highly significant correlation between hypertension and body mass index (BMI). Hence, the association between the two factors, viz., parental history of hypertension and BMI, among the young Indian male and female population was analyzed in this study.

Method: This cross-sectional study conducted in Central India comprised 100 young adults between 18 and 25 years of age. On the basis of parental history of hypertension, the subjects were divided into two groups: group 1 comprised youth with parental history of hypertension and group 2 comprised youth without parental history of hypertension, involving 32 and 68 subjects in each of these groups, respectively. Anthropometric measurements were done using standard methods, and BMI was calculated. Statistical analysis was done using Epi Info software.

Results: The study subjects in both groups were comparable in age. The mean age of the study participants was 19.32 years and 19.45 years in group 1 and group 2, respectively. The study did not find any association between BMI and parental history of hypertension.

Conclusion: The association between parental history of hypertension and BMI, the non-modifiable and modifiable independent risk factors, respectively, needs to be further explored in light of the iceberg phenomenon, multifactorial causation, and natural history of disease. It is worth mentioning that parental history of hypertension and other risk factors should be considered even if the BMI is normal as per Asian Indian cutoff levels.

## Introduction

Hypertension has a multifactorial etiology with functional and morphological abnormalities of vessel walls [1]. Genetic composition, age, dietary habits, level of physical activity, personality, and personal habits are epidemiological drivers of hypertension among adults.

It is noteworthy that parental history of hypertension (PHH) is a non-modifiable risk factor for developing hypertension in adult life. Environmental factors, viz., stress, lifestyle, occupation, habitation, geosocial factors, and economic status, also play a significant role [2]. Many studies have found higher arterial pressure in cases with a family history of hypertension [3]. Compared to the normotensive offspring of normotensive parents, the normotensive offspring of hypertensive parents had increased blood pressure and impaired arterial properties [4,5]. These differences are expressed conspicuously in men, but not in women [4]. Ambulatory blood pressure was associated with parental history of hypertension in men [6]. Cardiorespiratory phase synchronization is lower among those having non-hypertensive parents [7].

Obesity is a risk factor for hypertension. There is a significantly high correlation of hypertension with higher body mass index (BMI) among adolescents [8]. A longitudinal study found a positive correlation between the BMI of juveniles and their blood pressure in adult life [9]. Another study found no association between BMI and high-normal or elevated blood pressure in adolescent girls [10]. Higher BMI was in hypertensive as compared to normotensive children [8]. Hence, a cautious approach to understanding the pathophysiology and clinical aspects of hypertension for ascertaining the independent and collective role of various known and yet unknown extrinsic and intrinsic factors affecting and influencing the morbidity profile of an individual as per his/her genetic makeup is required.

Genetics and epigenetics have an important determinant role in the causation of the disease since the risk factors engrained in the genetic material get expressed later in life [1]. The presence of cardiac disease, stroke, diabetes, and obesity in parents is associated with various risk factors for disease development in their adult children [11]. Hence, the quantum and directionality of the multifactorial causation of disease have a strong basis of being rooted in the parental history of observed morbidities. Although the disease pattern among adolescents, youth, adults, and old age persons is undeciphered, the links of disease or related risk factors are prominently present in either parents or their ancestors.

The patterns of hypertension differ among various geosocial population settings, but the risk factors generally remain the same, triggering elements for the observance of hypertension at later stages of life [12,13]. Hypertension, one of the most common non-communicable diseases, still needs to be explored for the comprehensiveness of the combination of mechanisms enabling the body systems to become incapable of controlling the blood pressure within the known range of normal blood pressure. Demographic variables may be accentuating factors for environmental risks adversely affecting healthy living with lifestyle-induced hypertension and complications [2,8].

The association between BMI and parental history of hypertension among the young population requires closer scrutiny for an in-depth understanding of non-communicable diseases such as hypertension. Since parental history of hypertension and BMI are independent risk factors for hypertension, this study aimed to understand the interrelation between these two factors.

## Materials and methods

Study population

The study was conducted among 100 young adult males and females aged 18-25 in a tertiary healthcare center in Central India. The study was initiated after approval by the Institutional Ethics Committee of People's College of Medical Sciences and Research Centre, Bhopal, vide its letter number PCMS/OD/2017/1078 'D' dated 10/04/2017. The study followed the stated norms set by the institution for research studies.

Inclusion and exclusion criteria

The participants were selected per the inclusion criteria, which included the absence of any severe medical disorder, chronic illness, history of hospitalization of any duration during the last year, and history of any long-term medication. All participants gave informed written consent before the commencement of the study.

Anthropometric and other parameters

Information about age, sex, dietary habits, and residential location was recorded per the approved study format. Parental history was noted for hypertension, coronary artery disease, and stroke, as known to the study subjects. The history of hypertension in one or both parents was considered positive. The body weight of the study subjects was measured up to the 10th of a kilogram using an electronic weighing balance in light clothing and barefoot. Height was measured barefoot by a wall-mounted stadiometer in centimeters (cms). The body mass index (BMI) was calculated as weight in kilograms (kgs) divided by height in square meters (m^2^). The usual range of BMI was taken between 18 and 23 as per World Health Organization (WHO) criteria for Asians [14].

Categorization of study subjects

Based on the parental history of hypertension (PHH), the study participants were categorized as follows: group 1 having study participants with parental history of hypertension (with PHH) and group 2 comprising study participants without parental history of hypertension (without PHH). Parental history was taken as positive if one or both parents were confirmed hypertensive.

Statistical analysis

Data were thus collected and analyzed by relevant statistical tests using Epi Info software. Data are shown herein as mean, standard error (SE), and p-value. The level of significance was set at 0.05.

## Results

**Figure 1:**
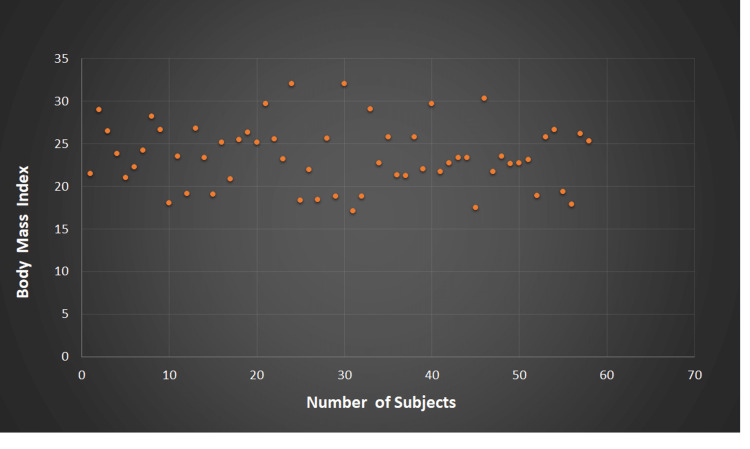
Body mass index among male subjects

**Figure 2:**
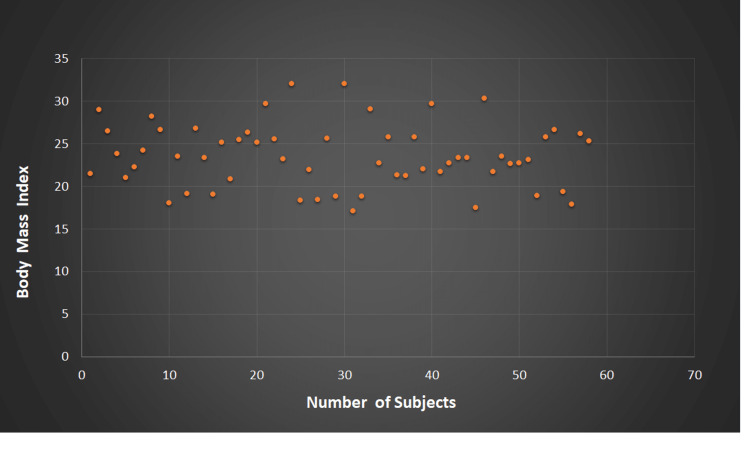
Body mass index among female subjects

The demographic and anthropometric profiles of the study subjects of both sexes are comparable since the age and body mass index (BMI) were non-significant (p>0.05) (Table 1). The mean age was 19.47 years for males and 19.33 years for females. The significant difference (p≤0.0001) of height (cm) and weight (kg) among male and female study subjects has no importance for comparability since the BMI derived from these datasets itself was non-significant (Figure 1 and Figure 2). Most subjects of these two groups were from urban areas and had active lifestyles. Of male and female subjects, 65% and 59% were vegetarian, respectively, and hence are also comparable for the present study.

**Table 1:** Anthropometric profile of male and female subjects (N=100) SE: standard error

Variables	Male (n_1_=58)	Female (n_2_=42)	SE	95% confidence level of mean difference	p
Age	19.47	19.33	0.266	-0.67, 0.39	0.5996
Height in cm (mean)	172.21	157.23	1.292	-17.54, 12.42	0.0001*
Weight in kg (mean)	69.99	55.52	2.174	-18.53, 9.91	0.0001*
Body mass index (mean)	23.60	22.46	0.761	-2.66, 0.37	0.0686
Lifestyle (sedentary/active)	2/56	0/42	-	-	-
Dietary habits (vegetarian/non-vegetarian/ovo-vegetarian)	38/15/5	25/12/5	-	-	-
Residence (urban/rural)	55/3	42/0	-	-	-

**Figure 3:**
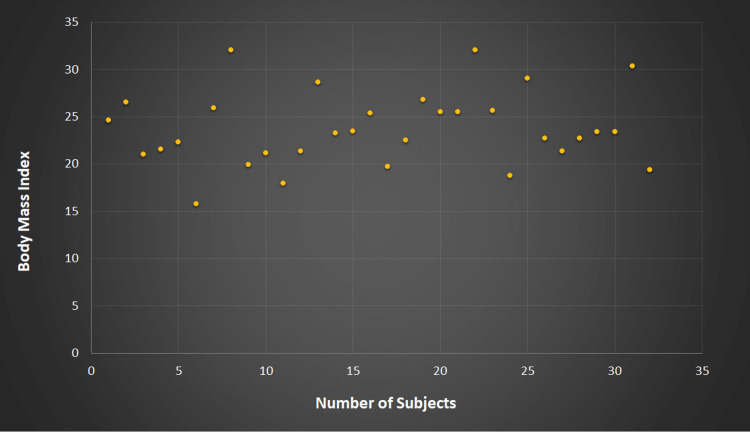
Body mass index in subjects with parental history of hypertension

**Figure 4:**
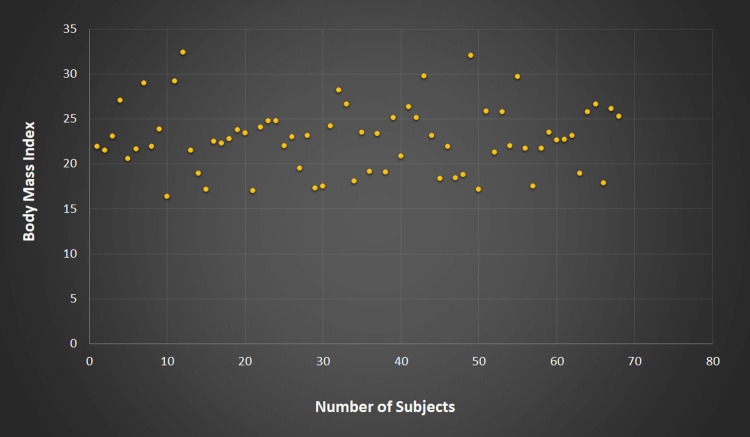
Body mass index in subjects without parental history of hypertension

The comparative study of various demographic and anthropometric observations expressed as mean±standard deviation among 32 subjects with PHH and 68 subjects without PHH showed no significant difference between the two groups for age, making these groups comparable (Table 2). Most study subjects in both groups were from urban areas and had an active lifestyle since all were youth, having a mean age of 19.32 years for those with parental history of hypertension and 19.45 years for those without parental history of hypertension. The distribution of BMI in both groups was comparable (Figure 3 and Figure 4).

**Table 2:** Association of anthropometric data of study subjects with and without parental history of hypertension (N=100) SE: standard error

Variables	With parental history of hypertension (n_1_=32)	Without parental history of hypertension (n_2_=68)	SE	95% confidence level of mean difference	P
Age (mean)	19.32	19.45	0.284	-0.43, 0.69	0.6487
Gender (male/female)	17/15	41/25	-	-	-
Height in cm (mean)	166.42	165.48	2.128	-5.16, 3.28	0.6597
Weight in kg (mean)	65.80	62.49	2.769	-8.77, 2.23	0.2405
Body mass index (mean)	23.76	22.82	0.818	-0.68, 2.55	0.1261
Lifestyle (sedentary/active)	0/31	2/67	-	-	-
Dietary habits (vegetarian/non-vegetarian/ovo-vegetarian)	17/9/5	46/18/5	-	-	-
Residence (urban/rural)	30/1	67/2	-	-	-

The BMI of males with PHH (n=17) and without PHH (n=41) was not significantly different. Similarly, the BMI of females with PHH (n=15) and without PHH (n=27) was not statistically different (Table 3).

**Table 3:** Body mass index of males and females with and without parental history of hypertension

Variables	With parental history of hypertension		Without parental history of hypertension		95% confidence level of mean difference	p
	n_1_	Mean	n_2_	Mean		
Body mass index (all)	32	23.76	68	22.82	-0.68, 2.55	0.1261
Body mass index (males)	17	24.51	41	23.22	-0.8637, 3.44	0.1176
Body mass index (females)	15	22.90	27	22.21	-1.8130, 3.19	0.2908

The dietary pattern was vegetarian in most of the subjects of the two groups and hence was comparable for the present study. The BMI of the two study groups showed no significant difference (p>0.05). Therefore, no association could be established between parental history of hypertension and BMI among the comparable groups of male and female young adults in the present study. Another noteworthy observation of this study is that the BMI in the group with PHH and among males in both groups is marginally above the Asian cutoff of normal BMI (Table 3).

## Discussion

Parental history of hypertension and obesity are independent risk factors for hypertension. The present study resonates with another research conducted among older adults, which found that BMI was significantly higher in those with a family history of diabetes mellitus, but not with a PHH [15]. Also, a study conducted at the University of Ferrara did not observe any significant difference in the BMI of the two groups of young adults with and without a family history of hypertension [16]. However, few studies have found significantly higher BMI in persons with PHH [3,17]. In a long-term follow-up study, 18-year-old females of hypertensive mothers, but not of hypertensive fathers, had a significantly higher BMI than females without a maternal or paternal history of hypertension [18]. It may be explained by the synergistic effect of two or more possible influencers on the causation of hypertension among persons with higher BMI and PHH. Unhealthy eating patterns in offspring with PHH may be confounding factors affecting BMI [19].

While comparing the geo-specific distribution of non-communicable diseases, Asians are at higher cardio-metabolic risk at a lower BMI than Europeans [14]. In a study among healthy children, the at-risk BMI range for hypertension was inversely proportional to PHH [20]. The obesity criteria for Asians were revised by the World Health Organization, given higher cardio-metabolic risk at lower BMI than Europeans [14]. The observation of marginally increased BMI above the normal Asian cutoff in the subjects with PHH in the present study is noteworthy in the Indian context. Hence, a high normal BMI should be interpreted cautiously in young adults with PHH. It is also pertinent that the preventive steps among individuals with higher BMI shall avert complications of hypertension. It is presumed to further revise the cutoff of BMI among Asians for the risk of hypertension among those with a PHH, which may be lower.

The prevalence of hypertension in youngsters ranges from 7% [21] to 25.1% [12] in the studies cited. This data is considerably high in comparison to the Northeast Asian country Japan where the prevalence is 0.6%-1.8% and 0.5%-0.7% in 15-17 years male and female participants and 3.2%-3.7% and 0.2%-0.4% in young adult male and female participants, respectively [13]. Being overweight and obese is associated with a higher risk of high blood pressure in younger and older children in India [12,22]. The rising prevalence of hypertension in children is closely associated with a striking increase in childhood obesity [22]. Thus, as a primary preventive measure, spreading awareness and implementing appropriate strategies to control weight at younger ages, especially in those with a PHH, become imperative.

This rise in known cases of hypertension cannot be explained by the tip of an iceberg phenomenon as the precursors and risk factors explain differences even in the clinical picture of hypertensives and their lifetime outcome in terms of morbidity and mortality profile [12]. It can be partially explained and justified by facility networks in government, private and unorganized sectors, increased awareness, and better-paying capacities among masses for healthcare services by mostly availing health insurance.

PHH can generally be an independent predictor for the causation of hypertension in adult life without consideration of BMI [23]. The genetic significance was illustrated by the finding of impaired renal handling of sodium in the proximal convoluted tubule in both obese and lean normotensive adolescent offspring of hypertensive parents as compared to those with normotensive parents [24]. Higher aldosterone levels were also reported in obese offspring with PHH compared to lean children [24]. Thus, although PHH is a predisposing factor for hypertension, the underlying mechanisms may vary from modifiable lifestyle factors to non-modifiable sodium renal sodium handling and other genetically acquired physiological mechanisms. Also, as increased BMI adversely affects renal sodium handling [24], it underscores the importance of keeping the BMI within normal limits. No difference between the two study groups herein attracts attention to explore various predictable factors for primary prevention and early detection of lifestyle diseases among youth.

Regional, racial, and local differences observed among various published studies were due to the difference in genetic factors, body constitution, dietary habits, personal preferences, lifestyle, habitation, and existing disease pattern in the concerned society [11,25]. However, a thread of uniformity, resonance, and reflectiveness always exists while assessing the underlying causes. BMI and PHH are almost equitably distributed among people of culturally and socially varying living standards. This is most likely due to the high preponderance and representativeness of major translational factors responsible for the noticeable observance of hypertension detected by chance during surveillance or by choice during existing diagnostic mechanisms in place.

Limitations of the study

The larger sample size and widespread geosocial distribution of study subjects could have enhanced the validity. In addition, the study format of the cohort would have been more conclusive as the larger study population is more likely to represent the study population. The results of this study apply to urban youth since most participants were from urban backgrounds. Separate studies need to be conducted for rural and tribal youth. The resource restrictions in the study setting essentially needed risk prediction and age-cumulative gender-wise stratification for cardiovascular risks based on observations of the study.

## Conclusions

Although the Asian cutoff for BMI is lower than the European as a health risk, the family history of hypertension needs to be explored cautiously among individuals with normal BMI, especially during screening and implementation of primary prevention measures for hypertension. Population-based strategic interventions are to be per the population-specific cutoff of BMI for achieving long-lasting desirable impact to bear public health gains for youth health. There is an observed need for conscious and cautious understanding-based identification of public health priorities on normality observance of BMI in the population and monitoring even the sub-normal BMI among those with a parental history of hypertension. A developmental model of change will go a long way in determining leadership in technology, innovation, and overall global governance. The resultant benefits of population strategy shall include economic gains in terms of the human development index, sustainable development goals, and national health priorities, which are likely to thrust momentum to enrich leadership in health program management, especially for non-communicable diseases, in the fast-changing world perspective resonating with geo-social, nutrition, and growth transition.

Having a normal BMI may not be considered a foolproof indicator of the nullified possibility of hypertension among Asians. Asian Indians with a normal BMI but with a family or parental history of hypertension must focus on other risk factors for hypertension. The present study is helpful in predicting the development of hypertension among genetically predisposed individuals. In conclusion, it would be highly beneficial to adopt effective measures during early life through primordial and primary preventive strategies enabling long-term sustainable positive health.
